# Survey of *Bordetella bronchiseptica* in Eastern Gray Squirrels (*Sciurus carolinensis*)

**DOI:** 10.1007/s10393-026-01788-4

**Published:** 2026-04-16

**Authors:** Gabriella Laird, Andrew Calinger-Yoak, Qirui Zhang

**Affiliations:** 1 Otterbein University, Westerville, OH USA; 2 Animal Disease Diagnostic Laboratory Ohio, Reynoldsburg, OH USA

**Keywords:** *Bordetella bronchiseptica*, kennel cough, eastern gray squirrels, real-time PCR, wildlife rehabilitation, ohio wildlife

## Abstract

Wild Eastern Gray Squirrels (*Sciurus carolinensis*) rehabilitated at a Midwestern facility, the Ohio Wildlife Center, were experiencing an increased mortality rate due to unknown respiratory illness in 2022–2023. A qPCR test was developed for *B. bronchiseptica* to test symptomatic animals. Though 65 individuals were tested, all tests were found to be negative for *B. bronchiseptica.* This negative result strongly indicates that the mortalities were caused by aspiration pneumonia or some other unknown pathogen.

## Introduction

*Bordetella bronchiseptica* is a gram-negative bacterium which causes a highly contagious and severe respiratory disease in multiple animal species (Szymczak et al., 2020). Commonly known as kennel cough, the pathogen causes severe inflammation of the trachea and bronchi and clinical signs may include coughing, sneezing, retching, nasal discharge, and pneumonia (Day et al., 2020). Many mammalian species, as well as humans in some cases, may contract kennel cough. It often results in tracheobronchitis in canines (*Canis lupus domesticus)* and atrophic rhinitis in swine *(Sus domesticus)* (Goodnow, 1980; Strausbaugh & Berkelman, 2003).

Additionally, there have been documented cases of *B. bronchiseptica* causing bronchopneumonia leading to mortality in red squirrels (*Sciurus vulgaris*) in Great Britain (Simpson et al., 2013). *B. bronchiseptica* may be responsible for undiagnosed respiratory illnesses in humans (Szymczak, et al., 2020). A live vaccine is widely available and commonly given as a core vaccine series in many small animal hospitals for domestic species. It is vital to closely monitor suspected outbreaks and contain the spread.

This study was originally developed to test Eastern Gray Squirrels admitted to a large wildlife hospital and rehabilitation center, the Ohio Wildlife Center (OWC). OWC staff observed symptoms of upper-respiratory disease, as well as increased mortality rates in their Eastern Gray Squirrel patients. These observations, as well as having a past history of *B. bronchiseptica* infection in a closely related squirrel species, led to the preliminary consideration that it was the cause of mortality because the highly contagious *B. bronchiseptica* can infect squirrels housed together.

## Methodology

### Testing Procedure Generation

We developed a testing protocol for *B. bronchiseptica* in partnership with the Ohio Animal Disease Diagnostic Laboratory (ADDL), using methods developed by Tizolova (Tizolova et al., 2014). They developed a more sensitive diagnostic assay for *B. Bronchiseptica* to assist human practitioners in differentiating between *B. Bronchiseptica* and *B. Pertussis.* The increased sensitivity (from 100 CFU/reaction to 24 CFU/reaction) created a more accurate test, which is why this procedure was selected as our primary reference (Tizolova et al., 2014). We selected and ordered the primers and probes from the commercial company Integrated DNA Technologies (IDT Website). The oligonucleotide sequences are listed in Table 1. The sensitivity and specificity of this test was confirmed using known positive and negative controls from ADDL reserves.

**Table 1:** Visual representation of the chosen primers and probes for *B. bronchiseptica* with their corresponding oligonucleotide sequence.

Species detected	Primers and probes	Oligonucleotide 5’-3’
*B. bronchiseptica*	Fla2	AGGCTCCCAAGAGAGAAAGGCTT
	Fla12	AAACCTGCCGTAATCCAGGC
	Fla-FAM3	ACCGGGCAGCTAGGCCGC

Reference strains of *B. bronchiseptica* were collected from the ADDL reserve and cultivated at 37±2°C for 24–48 hours on Tryptic Soy Blood Agar and MacConkey plates. For both the reference strains and suspected OWC samples, DNA was isolated using MagMax Core Nucleic Acid Purification Kit (Thermo Fisher, CA). Designed primers and probes were used for real-time PCR to amplify the intergenomic sequence between the *flaA* gene and the *fliA* gene. The PCR master mix consists of: 5 μl of TaqMan Fast Virus 1-step, 10.6μl of H2O, 1 μl of 10 μM Fla2, 1 μl 10 μM Fla12, and 0.4 μl10 μM fla-FAM3. 2 μl of the isolated DNA template was combined with 18 μl of the master mix for a total PCR reaction volume of 20 μl. Samples were cycled once using an Applied Biosystems 7500 Fast Real-time PCR System at conditions of 50°C for 5 minutes for Hold 1 (reverse transcription) and next 95°C for 20 seconds for Hold 2 (initial PCR activation). They were then cycled 45 times at conditions of 95°C for 3 seconds (denaturation) and 60°C for 45 seconds (annealing and extension). The fluorescence signal was collected at 60°C for each cycle (Tizolova et al., 2014). Known positive and negative control samples of *B. bronchiseptica* were included in each run.

### Sample Testing

Nasal swab samples were collected from Eastern Gray Squirrel individuals at the OWC hospital and tested over the course of 13 months (February 2023–February 2024). Squirrels are currently housed in small social groups of generally 3-8 individuals of a similar age. This is significant, as *B. bronchiseptica* infections are highly contagious. Samples were kept in saline and frozen at − 80°C until transportation. Samples were transported to the ADDL to be tested for *B. bronchiseptica* via qPCR.

### Serial Dilution and Specificity Test

Following the final batch of samples run for the OWC, a serial dilution was conducted using five cultured, known positives and one known positive lung tissue sample. The serial dilution was accomplished using a tenfold dilution technique.

We performed a specificity test for this qPCR method using a known positive sample of *B. bronchiseptica* with other non-target pathogens: *Bordetella avium, Bordetella hinzii, Bordetella petrii,* Bovine Respiratory Syncytial Virus (BRSV), Bovine Viral Diarrhea Virus (BVDV), Infectious Bovine Rhinotracheitis (IBR), Influenza A virus (IAV), Mycoplasma hyopneumoniae (MHP), Mycoplasma hyorhinis (MHR), Bovine Parainfluenza Virus Type 3 (BPIV-3), Porcine Parainfluenza Virus Type 1 (PPIV-1), Porcine Respiratory Reproductive Syndrome Virus (PRRSv), and Severe Acute Respiratory Syndrome Coronavirus 2 (SARS-CoV-2). By running the qPCR for *B. bronchiseptica* with a known positive, other species of the same genus, and other respiratory viruses, it can be made certain that the qPCR will not falsely test positive for a similar pathogen.

## Results

### Initial Testing

Sixty-five individuals with suspected symptoms from the OWC were tested via qPCR for *B. bronchiseptica*, and all samples were negative. *B. bronchiseptica* was confirmed to not be the cause of the increased mortality rates in Eastern Gray Squirrels seen by the OWC.

### Serial Dilution and Specificity Test

**Figure 1:**
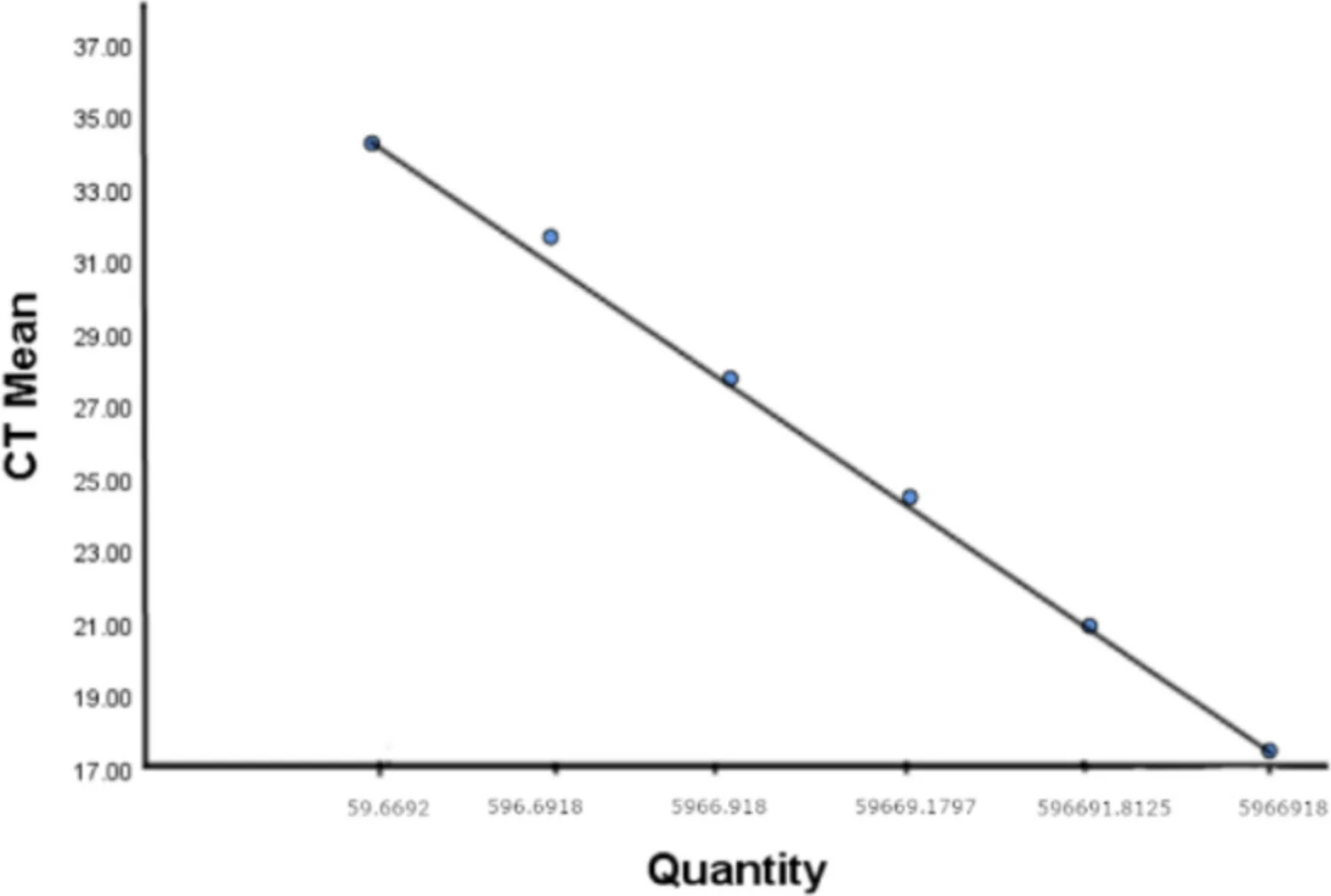
Standard curve for the tenfold serial dilution. The standard curve indicates that the PCR has an efficiency of 95% (slope − 3.435). CT mean represents the mean number of three threshold cycles for each sample, and the quantity represents the calculated number of molecules contained in each sample.

Our developed qPCR test was used to test the confirmed positive samples cultured by the ADDL, as well as a deceased rabbit that was sent to the ADDL for testing after dying of suspected respiratory illness. The qPCR for the rabbit presented a positive test result, providing indirect evidence. To confirm the sensitivity of the *B. bronchiseptica* qPCR test, a serial dilution was conducted. CT values and the number of DNA molecule copies were calculated and put into a standard curve (Fig. 1). An R^2^ of 0.995 was calculated alongside a slope of − 3.435 and Y-intercept of 40.8. The PCR efficiency percentage of 95% was found.

## Discussion

**Figure 2:**
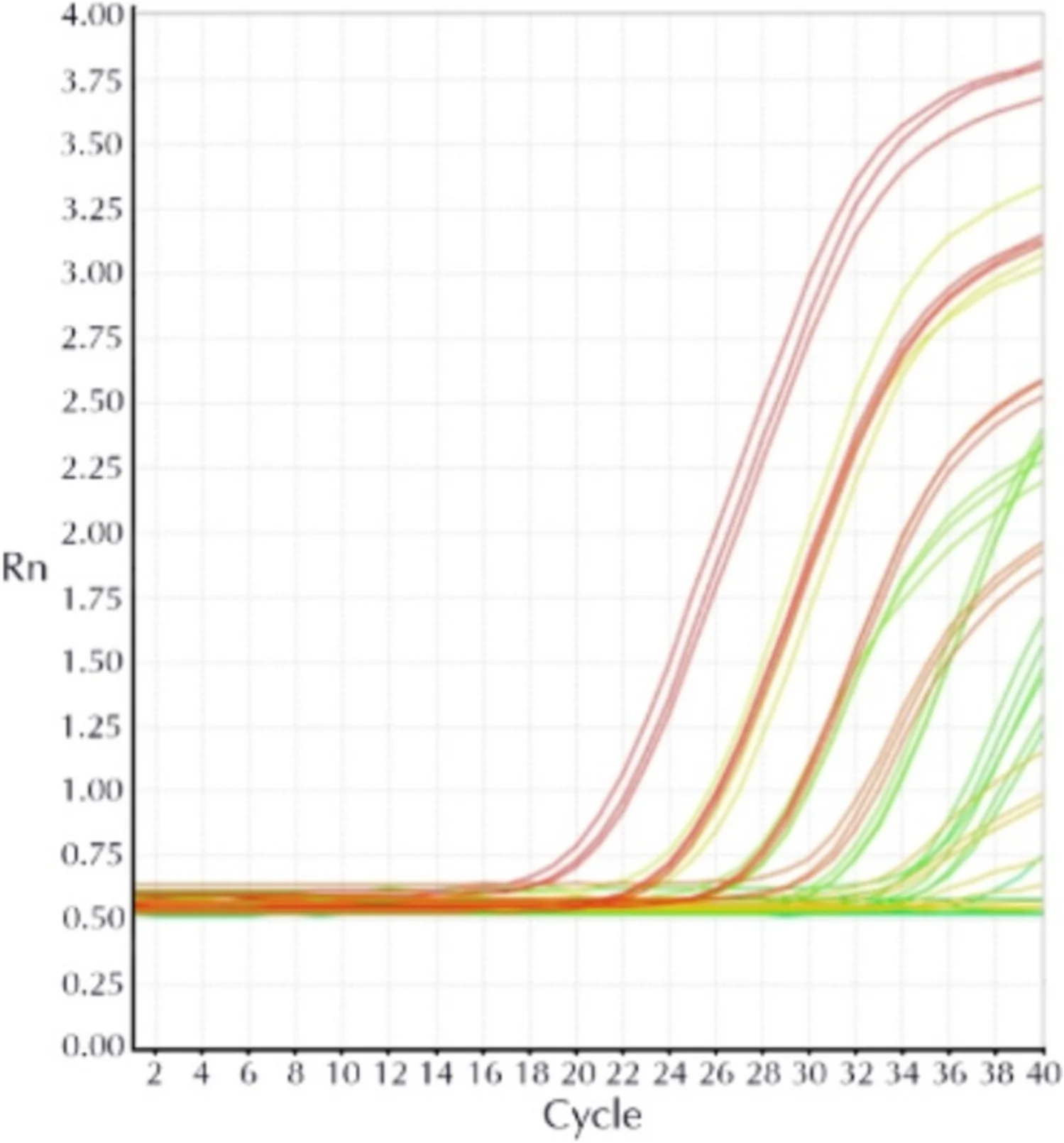
Amplification plot of the tenfold serial dilution. Rn represents the fluorescence dye normalized to the fluorescence signal (Ratio: reporter dye/passive reference dye). This is graphed by the number of cycles.

The initial symptoms of upper-respiratory disease and past history of *B. bronchiseptica* infection led to the preliminary consideration that it was the cause of mortality. As testing concluded that *B. bronchiseptica* was negative, it was unlikely to be the cause of this bout of respiratory illness, and thus, researchers and hospital staff have deduced the next most likely cause of the illness to be aspiration pneumonia (Fig. 2). The majority of the squirrels affected were between the neonatal and juvenile life stages, and aspiration pneumonia is a common ailment seen in wildlife rehabilitation, particularly in squirrels due to their voracious eating habits.

**Figure 3:**
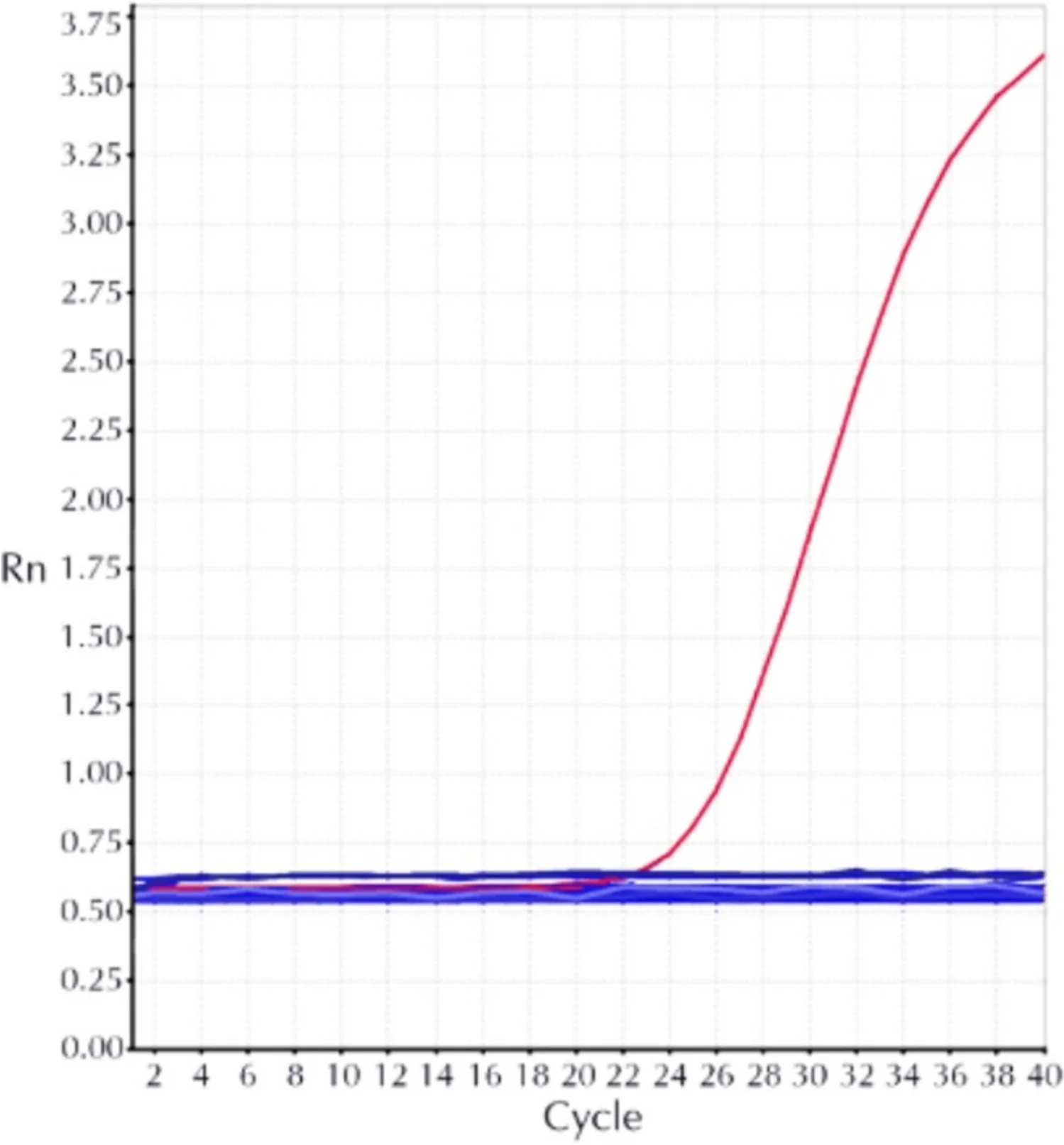
Amplification plot of the specificity test. A single known positive of *B. bronchiseptica* (shown in red) was run alongside samples of: *Bordetella avium, Bordetella hinzii, Bordetella petrii,* Bovine Respiratory Syncytial Virus (BRSV), Bovine Viral Diarrhea Virus (BVDV), Infectious Bovine Rhinotracheitis (IBR), Influenza A virus (IAV), Mycoplasma hyopneumoniae (MHP), Mycoplasma hyorhinis (MHR), Bovine Parainfluenza Virus Type 3 (BPIV-3), Porcine Parainfluenza Virus Type 1 (PPIV-1), Porcine Respiratory Reproductive Syndrome Virus (PRRSv), and Severe Acute Respiratory Syndrome Coronavirus 2 (SARS-CoV-2) (shown in blue).

Bacterial causes should be investigated further, including the common aspirational suspects *Haemophilus influenzae*, *Staphylococcus aureus*, *Streptococcus pneumoniae*, and anaerobes (Aspiration Pneumonia, 2009). We conducted a serial dilution to confirm the efficiency of the qPCR test. An efficiency of 95% was found, which allows the research team to have confidence in the results of this study. The specificity test was conducted to determine if the qPCR test would falsely test positive when presented with similar pathogens. To guard against cross-reactivity, the test was screened against available pathogens that could conceivably cause pulmonary involvement. The qPCR tested negative to all other pathogens aside from the known positive *B. bronchiseptica* sample (Fig. 3), indicating a low incidence of false positives.

The results of both necropsies were determined inconclusive by the ADDL pathology veterinarians. The OWC had two deceased squirrels frozen at − 80°C that were unlabeled with no demographic information. They were suspected to have died within the time period of the mortalities, so the researchers decided to go forward with necropsy to see if evidence of fluid could be found in the lungs. Aspiration pneumonia could also be confirmed via bacteria cultivation, but no new samples could be obtained. Necropsy is a less accurate way to confirm this diagnosis, but as the research team had no samples to cultivate, necropsy was decided, even lacking all of the standard background information.

## Conclusion

*B. bronchiseptica* is a prevalent pathogen in veterinary medicine, and due to its contagiousness, it is vital to prevent outbreaks whenever possible. While the OWC squirrels tested negative for *B. bronchiseptica,* the development of this test will streamline any necessary testing the ADDL may need to do in the future. This test will also be able to be run concurrently with other qPCR tests if desired. This will allow scientists to test for multiple infectious diseases simultaneously to quickly narrow down the cause of infection in an individual or outbreak. These test results were able to discount *B. bronchiseptica* as a cause of the mortalities, allowing the OWC to determine aspiration pneumonia as the next most likely cause. *B. bronchiseptica* has not been as thoroughly studied in wildlife as it has been in domestic species, and this study and testing protocol will hopefully be of use to other wildlife rehabilitators, conservationists, and scientists looking into cases of *B. bronchiseptica* or respiratory illness in wildlife. While this study focused on respiratory illness in eastern gray squirrels, this testing protocol may be used for any species to test for the presence of *B. bronchiseptica.* Other investigators dealing with young squirrel respiratory infection could refer to these results to inform their investigations into other possible pathogen outbreaks.
